# Sentiment Analysis of Social Media Users’ Emotional Response to Sudden Cardiac Arrest During a Football Broadcast

**DOI:** 10.1001/jamanetworkopen.2023.19720

**Published:** 2023-06-23

**Authors:** Nino Fijačko, Robert Greif, Gregor Štiglic, Primož Kocbek, Benjamin S. Abella

**Affiliations:** 1 Faculty of Health Sciences, University of Maribor, Maribor, Slovenia; 2 ERC (European Resuscitation Council) Research Net, Niels, Belgium; 3 Department of Anaesthesiology and Pain Medicine, Bern University Hospital, University of Bern, Bern, Switzerland; 4 School of Medicine, Sigmund Freud University Vienna, Vienna, Austria; 5 Faculty of Electrical Engineering and Computer Science, University of Maribor, Maribor, Slovenia; 6 Usher Institute, University of Edinburgh, Edinburgh, United Kingdom; 7 Faculty of Medicine, University of Ljubljana, Ljubljana, Slovenia; 8 Center for Resuscitation Science and Department of Emergency Medicine, University of Pennsylvania, Philadelphia

## Abstract

This case series analyzes social media users’ sentiments after successful cardiopulmonary resuscitation of Damar Hamlin following his sudden cardiac arrest on national television.

## Introduction

On January 2, 2023, over 23.6 million people witnessed the sudden cardiac arrest (SCA) of Damar Hamlin, a 24-year-old US football player, during a televised game. His collapse was promptly recognized as SCA; cardiopulmonary resuscitation (CPR) was provided. After defibrillation with an automated external defibrillator (AED), resuscitation was successful, and he was discharged home. After Christian Eriksen, a 29-year-old Danish football player, experienced SCA at the 2020 European Football Championship,^1^ Mr Hamlin’s resuscitation represented the second successful SCA resuscitation during a recent major sports event. In this case series, we analyzed social media users’ sentiments toward Mr Hamlin’s SCA and provide insight into the range of emotions spectators potentially experience witnessing SCA, with focus on whether his resuscitation prompted interest in CPR and AEDs.

## Methods

We did not follow any research guidelines or obtain institutional review board approval, as this case series did not involve patients or sensitive data. We compiled a database of all posts on Twitter containing prespecified hashtags from January 2 (8:55 PM EST) to January 4 (8:55 PM EST), 2023, using the academictwitteR package.^2^ Additionally, we collected posts from 6 hours before the event as baseline data. The following search query was used: #PrayersforDamar, #3, #DamarHamlin, #cardiacarrest, #CPRSavesLives, #Hamlin, #BuffaloBills, #emergencymedicine, #bengals, #Bills, #BillsVsBengals, #BillsNation, or #BillsMaf. We used the Syuzhet package^3^ to analyze the sentiment in posts. The National Research Council of Canada’s Word-Emotion Association Lexicon was used to analyze posts in 8 category emotions: trust, anticipation, joy, fear, surprise, sadness, anger, and disgust.^4^ Statistical analysis was conducted in R, version 4.1.0 (R Program for Statistical Computing) (eMethods in Supplement 1).

## Results

We included 83 065 posts that were posted within 24 hours of the event and evaluated changes in 13 367 posts that were posted during the next 24 hours. We used 2560 posts covering the 6-hour time window before the event to visualize the volume of posts aggregated in 8 emotion categories (Figure). Two waves of increased frequency of posts can be observed, with the first right after the event approximately 6 times higher than the next morning. In the first 24 hours, the most frequently expressed sentiment was anticipation (14 188 [17.1%]). Compared with 48-hour posts, changes in sentiment were most significant in anger, with a relative increase of 1.2% to 1372 posts (10.3%). With 5308 posts, *pray* was one of the most posted words in 24 hours (Table). Eight hundred and eighteen posts (1.0%) included hashtags #CPR, #AED, or #SuddenCardiacArrest, which decreased to 74 (0.6%) the day after Hamlin’s SCA. Those 818 posts contributed to each emotion sentiment category, where most ranged between 585 (71.5%) for sadness to 670 (81.9%) for trust, except anger with 88 (10.8%).

**Figure.**
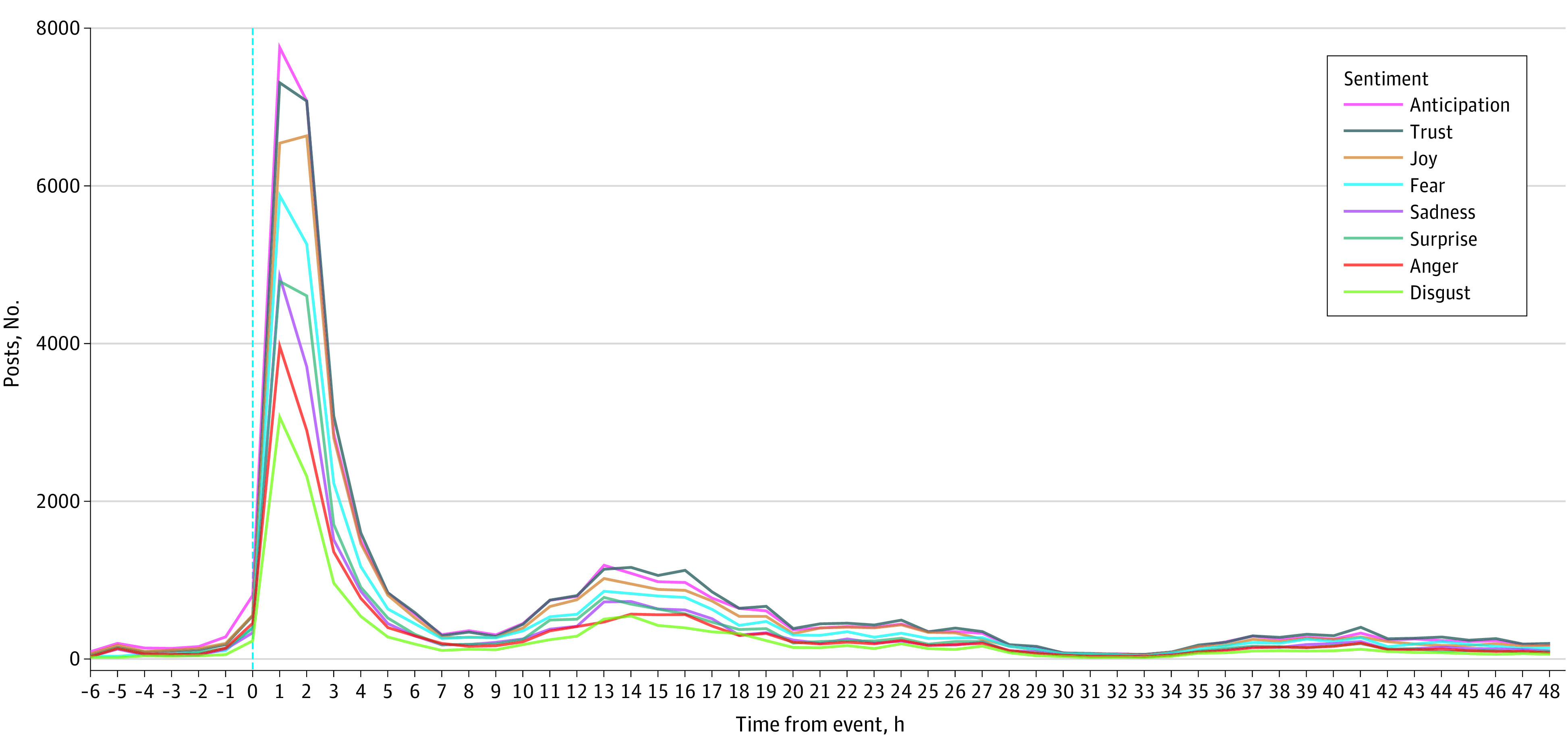
Frequency of Posts Aggregated in Emotion Categories Over Time

**Table.** Top 1000 Posts Based on 24- and 48-Hour Sentiment Analysis^a^

24-h Analysis		48-h Analysis	
Emotion sentiment category (No. [%] of posts)	The most frequent word in posts (No. of posts)	Emotion sentiment category (No. [%] of posts)	The most frequent word in posts (No. of posts)
Anticipation (14 188 [17.1])	Time (2529)	Trust (2320 [17.4])	Team (336)
Trust (14 148 [17.0])	Team (2279)	Anticipation (2290 [17.1])	Time (426)
Joy (12 419 [15.0])	Football (5887)	Joy (1969 [14.7])	Love (720)
Fear (11 400 [13.7])	Hamlin (10 603)	Fear (1822 [13.6])	ESPN (219)
Surprise (8773 [10.6])	Pray (5308)	Sadness (1391 [10.4])	Hamlin (859)
Sadness (8775 [10.6])	Cancel (1788)	Anger (1372 [10.3])	Shannon (292)
Anger (7574 [9.1])	Injury (1901)	Surprise (1296 [9.7])	Good (469)
Disgust (5787 [7.0])	Collapse (1296)	Disgust (907 [6.8])	Death (212)

^a^ Based on 83 065 posts for the 24-hour analysis and 13 367 for the 48-hour analysis.

## Discussion

Mr Hamlin’s successful resuscitation serves as an example of high-quality SCA resuscitation care. *Pray* and *hope*^1^ were the most used sentimental describing state verbs rather than action verbs, suggesting that laypersons are still relatively passive when thinking or hearing about SCA events. In the US, 90% of SCA events end in death.^5^ Public awareness campaigns during sports events can influence laypersons’ willingness to act when SCA occurs and encourage CPR training. Despite survival hinging on CPR and AED use, few posts highlighted these terms, and use of these terms decreased in the following 24 hours. This suggests a missed opportunity for public awareness of key resuscitation actions. The low frequency of these terms in public discourse may be related to the low national awareness of CPR and low prevalence of CPR training in the US, documented in prior work.^6^ This study is limited by the methodology used, as the sentiment analysis was only conducted on one social media platform and did not include posts and reposts in languages other than English. The current work suggests that there is a large opportunity to improve basic awareness of CPR and AED; partnership with sports leagues and celebrity figures who could champion the need for training may accomplish this.

## Notes

Supplement 1. **eMethods.** R Code

Supplement 2.
Data Sharing Statement
