# Supplementary material for: Sentiment Analysis of Social Media Users’ Emotional Response to Sudden Cardiac Arrest During a Football Broadcast
Source: JAMA Netw Open. 2023 Jun 23;6(6):e2319720. doi: 10.1001/jamanetworkopen.2023.19720 (PMC10290242; doi:10.1001/jamanetworkopen.2023.19720)
Supplement: Supplement 1. — eMethods. R Code [file jamanetwopen-e2319720-s001.pdf]

Supplementary Online Content

Fijačko N, Greif R, Štiglic G, Kocbek P, Abella BS. Sentiment analysis of social media users' emotional response to sudden cardiac arrest during a football broadcast. *JAMA Netw Open*. 2023;6(6):e2319720. doi:10.1001/jamanetworkopen.2023.19720

eMethods. R Code

This supplementary material has been provided by the authors to give readers additional information about their work.

© 2023 Fijačko N et al. *JAMA Network Open*.

eMethods. R Code

Note: below this is the R code used in the manuscript titled 'Sudden Cardiac Arrest during a Record-breaking Football Broadcast: Twitter Public Sentiment Analysis' by Nino Fijačko, Robert Greif, Gregor Štiglic, Primož Kocbek, and Benjamin S. Abella in JAMA Network Open. The code was working as of 18.04.2023, but might need modifications/updates if used in the future. It also needs a Twitter API Access Token from the Academic Research access.

```
#rm(list = ls())


assign("installP", function(pckgs){
  ins <- function(pckg, mc){
    add <- paste(c(" ", rep("-", mc+1-nchar(pckg)), " "), collapse = "");
    if( !require(pckg,character.only=TRUE) ){
      reps <- c("http://lib.stat.cmu.edu/R/CRAN","http://cran.uk.R-
    project.org");
      for (r in reps) try(utils::install.packages(pckg, repos=r),
    silent=TRUE);
      if(!require(pckg,character.only = TRUE)){   cat("Package:
    ",pckg,add,"not found.\n",sep="");
      }else{                                      cat("Package:
    ",pckg,add,"installed.\n",sep="");}
    }else{                                          cat("Package:
    ",pckg,add,"is loaded.\n",sep=""); } }
  invisible(suppressMessages(suppressWarnings(lapply(pckgs,ins,
    mc=max(nchar(pckgs)))))); cat("\n");
})

#might need to install dev version
#devtools::install_github("mjockers/syuzhet")

#ginstalling packages
installP(c("rtweet","academictwitteR","dplyr","ggplot2","tidytext","tidyr",
           "tm","tmap","wordcloud","syuzhet","openxlsx","data.table"))

#setting twitter academic token, included token at first use, par of
    package "academictwitteR"
#set_bearer()


#time of event
event_dt<-as.POSIXct("2023-01-03T01:55:00.000Z", tz="UTC", format= "%Y-%m-
    %dT%H:%M:%OSZ")

#2 days past event
time_till<-"2023-01-06"

#querry
twitter_query="#PrayersforDamar OR #3 OR #DamarHamlin OR #cardiacarrest OR
    #CPRSavesLives OR #Hamlin OR #BuffaloBills OR #emergencymedicine OR
    #Damar_Hamlin OR #bengals OR #Bills OR #Damar OR #BillsVsBengals OR
    #BillsNation OR #BillsMafia OR #prayfordamarhamlin"

#Note that initial run get all tweets in 2023 till about 2 days past event
get_all_tweets(query =twitter_query,
               start_tweets = "2023-01-01T00:00:00.000Z",
               end_tweets =paste0(time_till,"T00:00:00Z"),
```

© 2023 Fijačko N et al. *JAMA Network Open*.

```
             data_path = "data_tweets_DH/", bind_tweets = FALSE,
    n=10000000, export_query=TRUE, context_annotations = TRUE)

#update_collection(data_path = "data_tweets_DH/", end_tweets = "2023-01-
    05T01:55:00.000Z")

#creating a data.frame and saving
tweets <- bind_tweets(data_path = "data_tweets_DH/")
saveRDS(tweets,paste0("tweets_DH_before_after_event.rds"))

#loading tweets after getting them
#tweets <- readRDS("tweets_DH_before_after_event.rds")
tweets$days_event<-
    ceiling(as.vector(difftime(as.POSIXct(tweets$created_at,tz="UTC",
                                                        format= "%Y-%m-
    %dT%H:%M:%OSZ"),event_dt,units="days")))
tweets$RT<-"Tweet"
tweets$RT[grep("^RT",tweets[,"text"])]<-"Retweet"

#removing retweets
tweets_wc_no_retweet<-tweets[tweets$lang=="en",]


tweets_wc_no_retweet<-
    tweets_wc_no_retweet[setdiff(1:length(tweets_wc_no_retweet[,1]),

    grep("^RT",tweets_wc_no_retweet[,"text"])),]

#remove it from any character that you don’t want to show in your analysis
    such as hyperlinks, @ mentions or punctuations.
tweets_wc_no_retweet$text <-  gsub("https\\S*", "",
    tweets_wc_no_retweet$text)
tweets_wc_no_retweet$text <-  gsub("@\\S*", "", tweets_wc_no_retweet$text)
tweets_wc_no_retweet$text  <-  gsub("amp", "", tweets_wc_no_retweet$text)
tweets_wc_no_retweet$text  <-  gsub("[\r\n]", "",
    tweets_wc_no_retweet$text)
tweets_wc_no_retweet$text  <-  gsub("[[:punct:]]", "",
    tweets_wc_no_retweet$text)

#sentiment analysis

# Converting tweets to ASCII to trackle strange characters
tweets_no_retweet <- iconv(tweets_wc_no_retweet$text , from="UTF-8",
    to="ASCII", sub="")# removing retweets, in case needed
tweets_no_retweet  <-
    gsub("(RT|via)((?:\\b\\w*@\\w+)+)","",tweets_no_retweet )# removing
    mentions, in case needed
tweets_no_retweet  <-gsub("@\\w+","",tweets_no_retweet )
ew_sentiment<-get_nrc_sentiment(tweets_no_retweet)



Prepare_for_WC<- function(tweets, nrc_sentiment){
  tweets_wc_no_retweet_<-tweets
  ew_sentiment_<-nrc_sentiment

  sentim_vars<-c("anger", "anticipation", "disgust", "fear", "joy",
    "sadness", "surprise", "trust")

  for (i in 1: length(sentim_vars)) {
```

© 2023 Fijačko N et al. *JAMA Network Open*.

```
  tmp_text<-
  tweets_wc_no_retweet_$text[ew_sentiment_[,which(names(ew_sentiment_) ==
  sentim_vars[i])]>0]
  ifelse(i==1, list_sentim<-list(tmp_text),list_sentim<-
  c(list_sentim,list(tmp_text)))

}
names(list_sentim)<-sentim_vars


# convert each list content into a corpus and merge all documents into
  one single corpus
sentim_corpus <- lapply(list_sentim, function(x) VCorpus(VectorSource(
  toString(x) )))
sentim_corpus_all <- sentim_corpus[[1]]
for (i in 2:length(sentim_vars)) { sentim_corpus_all <-
  c(sentim_corpus_all, sentim_corpus[[i]]) }
# remove punctuation, numbers and stopwords
sentim_corpus_all<- tm_map(sentim_corpus_all, removePunctuation)
sentim_corpus_all<- tm_map(sentim_corpus_all, removeNumbers)
sentim_corpus_all <- tm_map(sentim_corpus_all, function(x)
  removeWords(x,stopwords("english")))

#removing some non-relevant words
words_to_remove <- c("said","from","what","told","over","more",

  "other","have","last","with","this","that","such","when",

  "been","says","will","also","where","why","would","today",

  "decorinspo","interiorandhome","kitchendecor","homeinspiration",

  "interior","bitch","homedeco","milf","decor","porn",".......bills")
sentim_corpus_all <- tm_map(sentim_corpus_all, removeWords,
  words_to_remove)

# compute term matrix & convert to matrix class --> you get a table
  summarizing the occurence of each word in each class.
document_tm <- TermDocumentMatrix(sentim_corpus_all)
document_tm_mat <- as.matrix(document_tm)
colnames(document_tm_mat) <- sentim_vars
document_tm_clean <- removeSparseTerms(document_tm, 0.8)
document_tm_clean_mat <- as.matrix(document_tm_clean)
colnames(document_tm_clean_mat) <- sentim_vars

#remove words in term matrix with length < 4
index <- as.logical(sapply(rownames(document_tm_clean_mat), function(x)
  (nchar(x)>3) ))
document_tm_clean_mat_s <- document_tm_clean_mat[index,]

#colSums(document_tm_clean_mat_s)


  #colSums(ifelse(document_tm_clean_mat_s>1,1,0))/length(document_tm_clean
  _mat_s[,1])

#colSums(document_tm_clean_mat_s!=0)/length(document_tm_clean_mat_s[,1])

#colSums(document_tm_clean_mat_s)/sum(document_tm_clean_mat_s)
```

© 2023 Fijačko N et al. *JAMA Network Open*.

```
  dimnames(document_tm_clean_mat_s)[[2]]<-
    paste0(dimnames(document_tm_clean_mat_s)[[2]], "\n(",

    round(100*colSums(document_tm_clean_mat_s)/

    sum(document_tm_clean_mat_s),1),"%)")

  tmp<- dimnames(document_tm_clean_mat_s)[[1]]
  dimnames(document_tm_clean_mat_s)[[1]]<-
    gsub("[\\.]{2,}","",dimnames(document_tm_clean_mat_s)[[1]])

  return (document_tm_clean_mat_s)

}

idx <-tweets_wc_no_retweet$days_event==1

wc_24_hours <- Prepare_for_WC(tweets_wc_no_retweet[idx,],
    ew_sentiment[idx,])


pdf("Figure_part_a_top_1000_24h_words_en.pdf", width = 5, height = 5)
comparison.cloud(wc_24_hours, max.words=1000, random.order=FALSE,c(4,0.4),
    title.size=1.2)
dev.off()

from_wc=c("injury","time","collapse","damarhamlin","football","cancel","pra
    y","team")

for(i in 1:8){
  print(paste0("Emotion: ", colnames(wc_24_hours)[i]," Word from WC: "
    ,from_wc[i], " Frequency: ",
    wc_24_hours[row.names(wc_24_hours)==from_wc[i],i]))

}


#tweets in the 24 hours after event
tweets_24h<-tweets[tweets$days_event==1 & tweets$lang=="en",]

tweets_24h$RT<-"Tweet"
tweets_24h$RT[grep("^RT",tweets_24h[,"text"])]<-"Retweet"

#looking for tweets with specific hastags in the 24 hours after event
idx<-which(unlist(lapply(tweets_24h$entities$hashtags, function(x)
    any(x$tag %in%
           c("CPR","AED","SuddenCardiacArrest","suddencardiacrrest") ))))

table(tweets_24h$RT[idx])
# Retweet   Tweet
# 264     818


table(tweets_24h$RT)
# Retweet   Tweet
# 233078   83065

# > 100*(818/83065)
# [1] 0.984771
```

© 2023 Fijačko N et al. *JAMA Network Open*.

```
idx <-tweets_wc_no_retweet$days_event==2

wc_24_48_hours <- Prepare_for_WC(tweets_wc_no_retweet[idx,],
    ew_sentiment[idx,])

length(tweets_wc_no_retweet[idx,1])*(colSums(wc_24_48_hours)/sum(wc_24_48_h
    ours))


pdf("Figure_part_a_top_1000_24h-48h_words_en.pdf", width = 5, height = 5)
comparison.cloud(wc_24_48_hours, max.words=1000,
    random.order=FALSE,c(4,0.4), title.size=1.2)
dev.off()

from_wc=c("shannon","time","death","espn","love","hamlin","good","team")

for(i in 1:8){
  print(paste0("Emotion: ", colnames(wc_24_48_hours)[i]," Word from WC: "
    ,from_wc[i], " Frequency: ",
    wc_24_48_hours[row.names(wc_24_48_hours)==from_wc[i],i]))

}


#tweets on day 2 after event (24-28hours after event)
tweets_day2<-tweets[tweets$days_event==2 & tweets$lang=="en",]
tweets_day2$RT<-"Tweet"
tweets_day2$RT[grep("^RT",tweets_day2[,"text"])]<-"Retweet"

idx<-which(unlist(lapply(tweets_day2$entities$hashtags, function(x)
    any(x$tag %in%

    c("CPR","AED","SuddenCardiacArrest","suddencardiacrrest") ))))

table(tweets_day2$RT[idx])
# Retweet   Tweet
# 198      74
table(tweets_day2$RT)
# Retweet   Tweet
# 55525   13367
# > 100*74/13367
# [1] 0.5536022

##analysis of tweets before and after events.. frequency with respect to
    sentiments

#saving for expedited access - run the following 2 commented lines first
    time
tweets_sentim <-
    cbind(timestamp=tweets_wc_no_retweet$created_at,ew_sentiment[,1:8])
saveRDS(tweets_sentim,paste0("tweets_DH_before_after_event_sentiment.rds"))

#tweets_sentim <- readRDS("tweets_DH_before_after_event_sentiment.rds")
tweets_sentim$hours_event<-
    ceiling(as.vector(difftime(as.POSIXct(tweets_sentim$timestamp,tz="UTC",
                                            format= "%Y-%m-
    %dT%H:%M:%OSZ"),event_dt,units="hours")))

data_ts<-melt(data.table(tweets_sentim), id.vars=c("hours_event"),
              measure.vars=c("anger", "anticipation", "disgust", "fear",
    "joy", "sadness", "surprise", "trust" ),
```

```
              variable.name="sentiment",value.name="value")

data_ts<-data_ts[,.(value=sum(value>0)), by=.(hours_event, sentiment)]

data_ts$sentiment<-factor(data_ts$sentiment,
                          levels=c("anticipation","trust", "joy",
    "fear","sadness", "surprise",   "anger", "disgust"))

#limiting the timerfame to 6 hours before event and 48 hours after event
data_ts<-data_ts[hours_event>=-6 & hours_event<=48]

pdf("Figure_sentiments_before_after_event_.pdf", height=8, width=18)
ggplot(data = data_ts, aes(x = hours_event, y = value, group = sentiment))
    +
  geom_line(linewidth = 2, alpha = 0.7, aes(color = sentiment)) + xlim(c(-
    6,48))+ylim(c(0,12500))+
  scale_colour_manual(labels=c("Anticipation","Trust", "Joy",
    "Fear","Sadness", "Surprise",   "Anger", "Disgust"),
                      values=c(
    "violetred4","goldenrod4","peru","dodgerblue4",  "midnightblue",
    "turquoise4","red4", "palegreen4")) +
  theme_minimal()+ geom_vline(xintercept=0, linetype="dashed", color =
    "red", size=1.05)+
  scale_x_discrete(limits=-6:48)+
    scale_y_continuous(breaks=seq(0,12000,2000))+
  xlab("Hours from event")+ylab("Number of tweets")+
  theme( legend.box.background = element_rect(fill = "white",color =
    "grey"),legend.position = c(0.93,0.75), axis.text.y = element_text(size
    = 16),
          axis.text.x = element_text(size =
    12),legend.title=element_text(size=24),
          legend.text=element_text(size=20),
    axis.title=element_text(size=20))+
  guides(color = guide_legend(title="Sentiment:",ncol = 1,override.aes =
    list(size = 4)))
dev.off()


data.table(tweets_sentim)[hours_event>=-6 & hours_event<0,.N]
#2560
#data.table(tweets_sentim)[hours_event>=-24 & hours_event<0,.N]

data.table(tweets_sentim)[hours_event>0 & hours_event<=2,.N]
#37155

idx<-which(unlist(lapply(tweets_wc_no_retweet$entities$hashtags,
    function(x)  any(x$tag %in%
      c("CPR","AED","SuddenCardiacArrest","suddencardiacrrest") ))))

tweets_cpr <-
    data.table(cbind(timestamp=tweets_wc_no_retweet$created_at[idx],ew_senti
    ment[idx,1:8]))

tweets_cpr$hours_event<-
    ceiling(as.vector(difftime(as.POSIXct(tweets_cpr$timestamp,tz="UTC",
                                                                format=
    "%Y-%m-%dT%H:%M:%OSZ"),event_dt,units="hours")))

#tweets_cpr[hours_event<0]
```

© 2023 Fijačko N et al. *JAMA Network Open*.

```
#tweets_cpr[hours_event>0 & hours_event<=24, lapply (.SD, function(x)
    sum(ifelse(x > 0, 1,0))),
#           .SDcols = c("anger", "anticipation", "disgust", "fear", "joy",
    "sadness", "surprise", "trust")]
#tweets_cpr[hours_event>0 & hours_event<=24, .N]
tweets_cpr[hours_event>0 & hours_event<=24, lapply (.SD, function(x)
    sum(ifelse(x > 0, 1,0))/.N),
            .SDcols = c("anger", "anticipation", "disgust", "fear", "joy",
    "sadness", "surprise", "trust")]
```

© 2023 Fijačko N et al. *JAMA Network Open*.
