# Supplementary material for: Sentiment Analysis of Social Media Users’ Emotional Response to Sudden Cardiac Arrest During a Football Broadcast
Source: JAMA Netw Open. 2023 Jun 23;6(6):e2319720. doi: 10.1001/jamanetworkopen.2023.19720 (PMC10290242; doi:10.1001/jamanetworkopen.2023.19720)
Supplement: Supplement 2. — Data Sharing Statement [file jamanetwopen-e2319720-s002.pdf]

Data Sharing Statement

Fijačko. Sentiment Analysis of Social Media Users' Emotional Response to Sudden Cardiac Arrest During a Football Broadcast. *JAMA Netw Open*. Published June 23, 2023. doi:10.1001/jamanetworkopen.2023.19720

Data
Data available: Yes
Data types: Other (please specify)
Additional Information: R code
How to access data: Supplement 1
When available: With publication

Supporting Documents
Document types: Statistical/analytic code
How to access documents: Supplement 1
When available: With publication

Additional Information
Who can access the data: Anyone.
Types of analyses: For any purpose.
Mechanisms of data availability: With investigator support.
Any additional restrictions: An investigator will need a Twitter API Access Token from the Academic Research access.
